# Chemotherapeutics as Immune Modulators for the Treatment of Triple-Negative Breast Cancer

**DOI:** 10.3390/cancers18101537

**Published:** 2026-05-09

**Authors:** Cory B. Fines, Sorcha Kelly, Helen O. McCarthy, Niamh E. Buckley

**Affiliations:** 1 School of Pharmacy, Queens University Belfast, 97 Lisburn Road, Belfast BT9 7BL, UK; 2 Radiation Oncology, University of Iowa, 375 Newton Road, Iowa City, IA 52242, USA

**Keywords:** triple-negative breast cancer (TNBC), chemotherapy, immunotherapy, lymphocytes, monocytes

## Abstract

Breast cancer is the second leading cause of cancer death in women. Among its various subtypes, triple-negative breast cancer (TNBC) is an aggressive form characterised by the absence of hormone receptor expression and lack of HER2 amplification, which severely limits treatment options. While some targeted therapies exist, chemotherapy remains the primary treatment for TNBC. Interestingly, despite TNBC patients showing the highest initial response rates to chemotherapy, they also experience the lowest overall survival. This phenomenon is often referred to as the “TNBC paradox,” which makes achieving curative outcomes incredibly challenging due to the unpredictable nature of the disease. This highlights a significant unmet clinical need to develop tailored medicine approaches to maximise patient response. Unlike other breast cancer subtypes, TNBC is considered “immune hot,” meaning it has a higher presence of immune cells within the tumour microenvironment. This characteristic has made the development of immunotherapies a major focus of research. PD-1/PD-L1 checkpoint inhibitors have already become the standard of care for high-risk early stage TNBC patients, and other immunomodulatory therapies, such as therapeutic vaccines, are currently under investigation. Given that chemotherapy is still the cornerstone of TNBC treatment, understanding its impact on systemic and tumour immune cells is crucial for improving the effectiveness of combination therapies. This review will delve into the specific roles of TNBC chemotherapies on key immune cells during both tumour progression and regression.

## Simple Summary

Triple-negative breast cancer is an aggressive form of breast cancer with limited treatment options, where chemotherapy remains the main approach. Although many patients initially respond well, long-term outcomes are often poor, highlighting a need for better strategies. This review explores how common chemotherapy drugs affect the body’s immune system, particularly within the tumour environment. Some treatments may help activate immune cells that fight cancer, while others may suppress them or have mixed effects. Understanding these immune-related effects can help guide personalised treatment approaches, ultimately improving survival in this difficult-to-treat disease.

## 1. Introduction

Breast cancer is the most common form of cancer and the second leading cause of cancer deaths in women [1]. Triple-negative breast cancer (TNBC) is an aggressive, invasive, and complex subtype of BC, characterised by the low/no hormone receptor expression (oestrogen (ERα) and progesterone (PR)) and lack of amplification of human epidermal growth factor receptor 2 (HER2) [2]. Due to the absence of these receptors, hormone and HER2 targeted therapies are ineffective in TNBC patients. Therefore, standard of care (SoC) treatment relies heavily on chemotherapy [3]. Common regimens including combinations such as 5-flourouracil (5-FU), epirubicin/doxorubicin (DOX) and cyclophosphamide (CTX) (often given together as the FEC cocktail) and/or taxanes (e.g., paclitaxel/docetaxel), and, in a small population, platinum-based agents (e.g., carboplatin or cisplatin) [2] (Table 1). TNBC exhibits a higher initial response rate to chemotherapy than other BC subtypes [4]; however, it has the lowest overall survival rate—a phenomenon known as the TNBC paradox [5]. Only 40–50% of TNBC patients achieve a complete pathological response (pCR) to neoadjuvant chemotherapy (NAC), leaving over half of patients facing drastically low odds of survival [3]. This disparity is caused by high heterogeneity and inherent chemotherapy resistance which leads to high rates of metastasis and recurrence. Due to this unpredictability in patient survival, there has been a push to determine which patients will respond to chemotherapy as well as develop new therapies for these patients. This has led to the investigation of the tumour microenvironment, and how different cells play a role in TNBC survival and response to chemotherapy.

The tumour microenvironment (TME) plays a critical role in TNBC progression, treatment resistance and patient outcomes [4]. Unlike other BC subtypes, TNBC tumours are more likely to be “hot” with higher levels of tumour-infiltrating lymphocytes (TILs) subtypes [5,6]. A systematic review showed that 20% of TNBC cases were classified as lymphocyte-predominant breast cancer (having over 50% TILs in stroma) compared to 6% for hormone receptor-positive breast cancer [7]. Different T-cells subpopulations can either be pro or anti-tumourigenic (Table 2). High levels of CD8+ cytotoxic T-cells are associated with increased survival in TNBC [8,9]. While there are conflicting results on the role of FOXP3+ T-regulatory-cell abundance on TNBC survival [10,11,12,13,14], it is typically seen that low levels of T-regulatory cells are favourable [15].

While T-cells are highly relevant in many cancers, there has been a growing interest in the role of monocytes in breast cancer (Table 2) [16]. Tumour-associated macrophages (TAMs) play a large role in the progression of breast cancer [17] and can account for up to 50% of the cell population in the TME of breast cancer patients [18]. TAMs are associated with worse disease-free survival (DFS) and overall survival (OS) rates [18,19,20,21,22]. TAMs can be classified based on their inflammation-promoting status into two distinct groups: alternatively activated, tumour-promoting TAMs also called M2-like TAMs, and classically activated, anti-tumorigenic or M1-like TAMs in the other [23]. However, this is an over simplified classification and, in reality, TAMs fall somewhere between these two groups [23]. M2-like TAMs promote cancer progression in many ways, including suppression of T-cell function via (i) depletion of necessary metabolites for T-cell proliferation such as L-arginine via the production of Arginase 1 (ARG 1), (ii) producing anti-inflammatory cytokines, including IL-10 which induces T-regs, and (iii) activating the T-cell checkpoint blockade (PD-1/PD-L1) [23]. Meanwhile, M1-like macrophages can produce pro-inflammatory cytokines, activate CD8+ T-cells and can kill tumour cells [23]. Examples of pro inflammatory cytokines include Interleukin 17 (IL17), Interferon Gamma (IFN-γ), Tumour Necrosis Factor Alpha (TNFα), inducible Nitric Oxide Synthase (iNOS), IL6, IL8 and CCR7, while anti-inflammatory cytokines include IL4 and IL10.

This highlights the potential for the TME to positively or negatively impact patient outcomes which has driven the development of immunotherapies for TNBC. After success in other cancers [24], the programmed death ligand 1 (PD-L1) immune checkpoint inhibitor (ICI), Atezolizumab, was fast track approved for locally advanced and metastatic TNBC patients. However, it was then withdrawn after further trials showed no significant benefit to overall survival when using Atezolizumab. The programmed cell death protein 1 (PD-1) inhibitor, pembrolizumab, was then tested and approved and is now the standard of care, in combination with chemotherapy, for the treatment of high-risk, early stage, PD-L1-positive TNBC patients [25]. While the use of immunotherapies and other targeted treatments such as Trodelvy has grown in interest, the use of chemotherapy is still essential, and will be likely be used in combination with any existing and/or new immunotherapies for the foreseeable future. Therefore, in this review, the immunomodulatory role of TNBC relevant chemotherapies is explored given the important role that TME plays in TNBC prognosis and tumour progression, as well as their potential to positively or negatively impact immunotherapy response.

## 2. Impact of Chemotherapy on T-Cells

### 2.1. Doxorubicin (DOX)

As previously mentioned, TNBC is known to have a very heterogenous response to NAC, where some patients achieve a pCR (responders) and others do not, demonstrating residual disease (non-responders) [3]. DOX’s impact on the immune system was compared in a 4T1 DOX-sensitive and DOX-resistant model, modelling responders and non-responders respectively [26]. Balb/c mice were orthotopically inoculated with 4T1 cells and once the tumour reached 100 m^3^, they were treated with 5 mg/kg DOX I.V. three times over 6 days. Mice with tumours that were comparable to untreated tumours were deemed DOX-resistant, and those that decreased in tumour volume were deemed DOX-sensitive. DOX-sensitive tumours had increased CD3+ T-cells compared to untreated and resistant tumours. Interestingly, while there was no difference in CD4+ cells between the groups, the resistant tumours had significantly more CD8+ T-cells compared to sensitive tumours, while the sensitive tumours had a larger population of γδ IL-17+ T-cells [26].

A 4T1 mouse breast cancer orthotopic tumour model was used to investigate the role of various TNBC standard of care chemotherapies on T-cell infiltration [27]. Liposomal DOX (DOXO-cell, 7 mg/kg), PTX (22.5 mg/kg) and combination PTX (10.35 mg/kg) + carboplatin (40 mg/kg) were given intravenously (I.V.) and organs harvested 48 h later and analysed by IHC. DOX was the only treatment that led to an increase in not only the CD3+ T-cells but also the CD8+ cytotoxic T-cells in the tumour stroma [27]. While no treatment decreased FOXP3+ T-regs, DOX did increase the CD8+/CD3+ ratio while also decreasing the FOXP3+/CD3+ ratios, indicating an increased pro-inflammatory TME. In another orthotopic 4T1 study, mice were treated with either 2.5 mg/kg or 5 mg/kg I.P. of DOX on day 7 and 12 and tissues were collected on day 14, 17 and 23 [28]. Both CD4+ and CD8+ T-cell abundance increased in the blood and spleen in a dose-dependent manner. This increase in systemic T-cells only lasted 5 days after treatment, where levels became the same as untreated mice 10 days after treatment.

### 2.2. Taxanes

While PTX showed no effect on T-cells in a 4T1 model when used as a single-agent [27], another study showed that paclitaxel micelles (POx-PTX) had an immune effect in 4T1 tumour-bearing mice. Mice were treated with 30 mg/kg of POx-PTX I.V., and tissues were harvested 72 h after the second dose. After treatment, there was an increase in CD4+ and CD8+ cells in the tumour while the T-reg abundance did not change [29]. Unfortunately, this paper did not have paclitaxel as a control; however, the results could be different to single-agent PTX due to the increased dosage and nano benefits of the micelle formulation.

Another study treated 4T1 orthotopic tumour-bearing mice with DOX (4 mg/kg) in combination with PTX (5 mg/kg) in the intraperitoneal cavity (I.P.) twice a week for 2 weeks [30]. While the time from last treatment to organ collection is unclear, an increase in the abundance of CD3+ T-cells was seen as measured by flow cytometry. More specifically, treatment reduced the amount of CD4+ TNFR2+ FOXP3+ T-regs in the tumour while increasing the abundance of CD8+ TNFR2+ subset. In the spleen, chemotherapy increased the CD3+ population, seen with a slight increase in both CD4+ and CD8+ populations, while reducing T-regs [30]. This suggests that combination therapy can and should be optimised for the best immune response and tumour reduction.

### 2.3. 5-Fluorouracil (5-FU)

In a 4T1 TNBC orthotopic mouse model, 5-FU was given three times a week for 3 weeks at either 5, 10, 25, or 50 mg/kg (I.P.) [31]. 5-FU did not significantly impact T-regs at any dose or cycle. However, there was one anomaly where 10 mg/kg saw a large increase in T-regs after three cycles. This seemingly random increase could be due to biological variability; however, the authors did not address these changes [31].

Due to a lack of literature on 5-FU in TNBC, the role of 5-FU in the immune system was expanded to other cancers. In a CT26 colon cancer mouse model, 5-FU (40 mg/kg) I.P. was administered for 4 cycles. 5-FU was given every other day for three total injections during cycle 1 and given every day for 4 days for cycles 2–4. The immune populations were assessed 7 days after the last treatment of the first and third treatment cycle [32]. No changes in splenic immune populations were seen after one treatment cycle, while a significant decrease in splenic CD4+ T-cells and B-cells was seen. In the tumour, there was a significant increase in CD8+ T-cells after one treatment cycle. Meanwhile there was increase in CD45-PD-L1+ (non-immune cells) after both one and three treatment cycles, possibly indicating repression of anti-tumour immune functions which could lead to relapse after ending 5-FU treatment [32]. When blood samples were compared from colon cancer patients who had received 5-FU and those who had not, it was shown that patients that receive 5-FU had increased circulating CD8+ levels [33].

### 2.4. Cyclophosphamide (CTX)

In a 4T1 TNBC orthotopic mouse model, CTX dissolved in drinking water, at approximately 5, 10, 20 or 40 mg/kg showed no change in T-regs, however B-cells were reduced at higher doses [31]. Again, due to a lack of literature on CTX in TNBC, the role of CTX in the immune system was expanded to other cancers. In a colon cancer rat study, various cytotoxic agents were explored for their role on T-regs (CD25+) including DOX, 5-FU and CTX. Seven days after a single dose of single-agent chemotherapy, only CTX decreased the CD4+ CD25+/CD4+ splenic T-cell ratio, indicating a decrease in T-regulatory cells [34]. The fact that DOX did not affect T-regs, while aforementioned studies showed DOX did decrease T-regs in a TNBC mouse model, demonstrates that the role of DOX is tumour dependent.

This led to a clinical trial in which the efficacy of CTX in reducing T-regs was tested in patients with metastatic cancer, including three patients with breast cancer (France Trial #2003/19). However, CTX showed no impact on the reduction in T-regs [35], which is in contrast to another trial where CTX did decrease T-regs in metastatic cancer patients [36].

### 2.5. Gemcitabine (GEM)

Gemcitabine (GEM) has been thoroughly explored in pancreatic cancer, as it is the standard of care [37]. Pancreatic patients given GEM saw insignificant changes in CD4+, CD8+ and B-cells, and GEM was deemed to not be immunosuppressive [38]. Moreover, multiple studies in pancreatic cancer patients have shown GEM is able to reduce the T-reg population [39,40,41,42], which are correlated to tumour progression and a worse overall survival, again supporting the fact that GEM promotes an immunocompetent environment. In terms of TNBC, splenic lymphocytes from untreated and GEM-treated (60 mg/kg I.P.) 4T1 bearing mice were collected and expanded in in vitro culture. Tumour-bearing untreated mice had less T-cell proliferation compared to non-tumour-bearing mice [43]. GEM-treated tumour-bearing mice restored T-cell proliferation, having a greater expansion of T-cells compared to untreated mice as well as having a greater IFN-γ response [43]. In a 4T1 TNBC mouse study, GEM (60 mg/kg I.P.) was given to 4T1 subcutenous (flank) tumour-bearing mice, and the immune response was measured 24, 48 and 96 h after the third treatment [44]. It was found that GEM increased CD3+ abundance in both the blood and spleen, while decreasing CD8+, CD4+ and FOXP3+ T-cells in the tumour. In our own studies, GEM (60 mg/kg I.P.) was given to 4T1 orthotopic (mammary fat pad) tumour-bearing mice and assessed 24–96 h after treatment [45]. After 24 h an increase in tumour and spleen CD3+ cells was reported while no change in CD4+ or CD8+ was seen. Both studies concluded that the immune response was acute, with the greatest effect seen at 24 h and then diminishing over time. Furthermore, when the dose of GEM was lower (30 or 15 mg/kg), there was a dose-dependent decrease in the increase in CD3+ cells in the spleen.

### 2.6. Immune Checkpoints

With pembrolizumab being commonly used for TNBC as well as investigations into other ICIs, we wanted to highlight the impact chemotherapy has on immune checkpoint markers. 4T1 TNBC cells were treated with DOX in vitro, and 24hrs later were stained with antibodies including PD-L1 [46]. There was an increase in PD-L1 expression in a dose-dependent manner. Similarly, MDA-MB-231 cells were treated with either DOX, EPI, PTX or docetaxel for 4 or 24 h and then the change in PD-L1 protein was assessed via Western blot [47]. While EPI, PTX and docetaxel increased PD-L1 protein levels at 4 and 24 h, DOX did not increase PD-L1 levels. However, when another TNBC cell line was treated, Hs578T, neither of the three chemotherapies increased PD-L1 expression [47]. Furthermore, 14 lesions in 13 TNBC patients who received anthracycline and/or taxane-based NAC were assessed for PD-L1 expression change. Only four cases of PD-L1 change were observed. Two cases had an increase in expression, one case had a positive conversion (no expression to expression) and one case a negative conversion (expression to no expression) [47]. In a 4T1 model, Liposomal DOX (DOXO-cell, 7 mg/kg), PTX (22.5 mg/kg) and combination PTX (10.35 mg/kg) + carboplatin (40 mg/kg) were given intravenously and changes in PD-1, T-cell immunoglobulin and mucin domain-3 (TIM-3) and cytotoxic T lymphocyte-associated protein-4 (CTLA-4) gene expression were measured [27]. All three drugs reduced PD-1 expression, while DOX was the only drug to significantly reduce TIM-3 and show a trend in reducing CTLA-4. Meanwhile PTX and PTX+ carboplatin show a trend towards increasing CTLA-4 gene expression. The role of chemotherapy on these markers, and the expression of these markers on chemotherapy response is not straightforward. In a study of 177 early stage breast cancer patients treated with NAC, it was found the patients with high expression of PD-1 and PD-L1 had significantly higher rates of TNBC and higher rates of non-pCR [48,49]. The expressions of PD-1 and PD-L1 were associated with a significantly deceased DFS in TNBC [48]. However in a meta-analysis with a total of 2403 non-metastatic TNBC patients, the expression of PD-L1 [50] was associated with better chemotherapy response rate and better DFS and OS than patients that were PD-L1-negative. This better response to chemotherapy was also seen with CTLA-4-expressing patients [51]. However, the expression of TIM-3 was associated with a worse chemotherapy response in TNBC patients [51]. While the expression status roles on chemotherapy response are complex, it is more straightforward for ICIs. TNBC patients that were positive for PD-L1 had improved OS from ICIs while PD-L1-negative patients had no significant improvement [52]. As will be a trend throughout this review, the role of chemotherapy on the immune profile and whether it is beneficial is complex and context dependent.

## 3. Impact of Chemotherapy on Macrophages

As previously mentioned, TAMs are associated with a worse prognosis in TNBC [18,19,20,21,22]. Due to the prevalence of macrophages in TNBC, there is a growing interest in how chemotherapies impact TAMs abundance and polarisation [53]. When treating tumours, chemotherapy can play both a direct and indirect role on the immune cells in the tumour, spleen and blood. The following studies try and tease out the direct effect of chemotherapy on macrophages as well as indirect by using media transfer studies from treated cancer cells.

### 3.1. Doxorubicin (DOX)

There is limited data on the role of DOX on TAMs. One study showed DOX was able to kill THP1 cells in a short-term MTT assay [54]. Another study showed that DOX-treated RAW 264.7 macrophages had no effect on nitric oxide (NO) production or iNOS, TNFα, IL6 or TLR4 mRNA expression; therefore, it was concluded DOX alone is not capable of stimulating macrophages [55].

### 3.2. Taxanes

There is limited data on the role of taxanes on TAMs. Early studies showed that PTX has similar properties to LPS in inducing macrophages to release NO, IL1 and TNFα and play a role in its anti-tumour effect [56,57,58,59]. However, this was not seen in a study on breast cancer patients. Serum from 30 breast cancer patients was taken before treatment and after the last treatment cycle of either single-agent PTX or docetaxel [60]. All patients responded to therapy and experienced an increase in serum IFN-γ, IL-2, IL-6 and GM-CSF and a decrease in IL1 and TNFα cytokine levels.

Another study isolated monocytes from heathy donors and treated them with either PTX or docetaxel [61]. Docetaxel increased generation of M1-like macrophages while PTX had no effect on polarisation.

### 3.3. Gemcitabine (GEM)

To our knowledge there are currently only two studies looking at changes in macrophages in breast cancer. In a 4T1 subcutaneous model, GEM-treated mice (60 mg/kg I.P.) showed a decrease in circulating and splenic macrophages (CD45+ CD11b+ F480+ Ly6C-) but no decrease in tumour macrophages. There also appeared to be a decrease in M2-like macrophages defined by a decrease in Arg1+ macrophages in the tumour [44]. In our own 4T1 orthotopic model, GEM (60 mg/kg I.P.) increased splenic macrophage abundance (CD45+ CD11b+ F4/80+ and CD45+ CD11b+ F480+ Ly6C-) while simultaneously decreasing tumour macrophage abundance. Again, these changes were greatest at 24 h and with 60 mg/kg compared to 30 and 15 mg/kg [45].

While there is limited breast cancer data, there have been other studies investigating GEM’s role on macrophages. Mouse peritoneal macrophages (PMs) were collected from C57Bl/6 mice, and these unpolarised M_0_ PMs were then treated with GEM and polarisation analysed via PCR [62]. GEM was able to increase M1-like markers TNFα and iNOS. IL4 was used to polarise PMs into the M2-like state, where they then were then treated with IL4 again in the presence of GEM. GEM prevented IL4’s reduction in M1-like markers and increase in M2-like markers. This was also seen in a human macrophage cell line (THP1), where GEM increased CD80, CCR7, IL6 and IL8 and decreased CD163 and CD206 gene expression.

However, in contrast, GEM was shown to increase M2-like macrophages in a pancreatic mouse tumour model [63]. These results were confirmed in vitro when pancreatic cells treated with GEM resulted in IL8 induction and the subsequent migration, invasion, and growth of macrophages [63]. Furthermore, after treatment with GEM, pancreatic cell conditioned media was transferred to mouse macrophage RAW264.7 cells, which in turn increased M2-like protein expression [63]. Similar results were seen in a pancreatic patient-derived xenograft mouse model where after I.P. treatment of GEM there was an increase in M2-like macrophages in the tumour. Furthermore, GEM selectively decreased pathways necessary for M1-like macrophage ATP production, specifically the rate-limiting enzymes for glycolysis (Idh3a, Idh3b, Idh3g, Idh1, Idh2) [64].

In our own studies we saw mixed results. We used THP1 cells to investigate the ability of GEM to polarise macrophages both directly and indirectly in vitro. After differentiating the THP1 cells into macrophages (D-THP1) they were exposed to GEM for 24 h. An increase in both M1- (CD80, IL1B and TNFα) and M2-like (MRC1, Dectin1 and DCSign) genes were upregulated. Indirect polarisation was measured by treating 4T1 cells with GEM then transferring the media to the THP1 cells. Again, we saw an increase in both M1- and M2-like genes. Therefore, as seen throughout all these studies, GEM seems to create a mixed macrophage phenotype. However, we propose that decreasing the overall abundance of macrophages in tumours could outweigh the necessity of GEM to skew macrophages into a M1-like state.

### 3.4. Platinum Agents

While platin-based chemotherapies are mainly reserved for BRCA-mutated TNBC patients, the studies performed are of great interest as they may be expanded to other TNBC chemotherapies.

The direct and indirect role of cisplatin on macrophages was studied. Media from MCF-7 ER+ breast cancer cells was shown to polarise human monocytes into the M2-like state. When these macrophages were then treated with cisplatin, an increase in pro-inflammatory cytokines such as TNFα and IL8 was observed [65]. Conversely, in another study, the indirect role of platins was studied. Ovarian and cervical cancer cells were treated with cisplatin and carboplatin, and the subsequent media was transferred to monocytes, where an increase in M2-like markers was observed [66].

Carboplatin on the other hand showed an increase in M1-like markers in vitro using THP-1 cells as well as PBMCs that had been differentiated into macrophages [67]. Macrophages directly treated with carboplatin showed an increase in M1 markers and a decrease in M2 markers. Similar results were also seen in an indirect manner, where carboplatin-treated ovarian cancer cells can help maintain macrophage CD80 expression and decrease CD206 expression. The effects of carboplatin were then observed in an ovarian mouse model where CAOV4 human ovarian cancer cells were injected I.P. in immunodeficient nude mice (NU/J) mice and which were treated with I.P. carboplatin (50 mg/kg) four times once a week. Tumours were collected 3 days after the last treatment, where a reduction in both M1 and M2 macrophages in the tumour was seen [67].

## 4. Impact of Chemotherapy on Myeloid-Derived Suppressor Cells

Another important myeloid-derived cell type in TNBC are myeloid-derived suppressor cells (MDSCs). Bone marrow progenitor cells typically undergo myelopoiesis to differentiate into mature granulocytes, monocytes, dendritic cells, or macrophages. However, tumour progression can inhibit this differentiation, leaving a large immature heterogeneous population of activated MDSCs [68]. Similar to TAMs, the abundance of MDSCs in the peripheral blood of breast cancer patients has been associated with tumour stage [69], with the highest levels present in those with metastatic disease [70] and in the TNBC subtype [71]. Patients who achieved pCR to neoadjuvant chemotherapy had lower baseline levels of MDSCs than those who did not achieve pCR [72]. Classically, MDSCs have been defined as CD11b+ GR1+ in in vivo mice studies and variable responses have been seen depending on chemotherapy used, frequency and in what tumour model.

### 4.1. Gemcitabine (GEM)

Gemcitabine was the first chemotherapy to demonstrate ability in reducing MDSCs. In a 2005 study, GEM was shown to be able to reduce MDSCs as measured by flow cytometry in a TC-1 subcutaneous lung cancer mouse model [73]. In 2009, this reduction in MDSCs after GEM treatment was also reported in a 4T1 breast cancer model [43]. GEM (60 mg/kg I.P.) was able to reduce MDSCs both after a single dose in larger tumour (>60 mm^3^) and multiple doses starting 5 days after inoculation [43].

### 4.2. Doxorubicin (DOX) and 5-Fluorouracil (5-FU)

The ability of GEM to reduce MDSCs both in spleen and tumour increased curiosity in other chemotherapy agents. A 2010 study used an EL4 thymoma mouse model to investigate the efficacy of different cytotoxic agents in reducing MDSCs including cyclophosphamide, doxorubicin, oxaliplatin, paclitaxel, 5-FU and raltitrexed [74]. DOX and 5-FU were able to reduce MDSCs in both the spleen and tumour while other agents had no effect or actually increased MDSC levels [74]. The reduction in MDSCs after 5-FU treatment was also seen in the tumour and bone marrow of a CT26 colon tumour mouse model [33]. This was followed up by taking blood samples of cancer colon patients, where a reduction in MDSCs was seen in patients who had received 5-FU compared to those who did not [33]. In a B16/F10 melanoma mouse model, with additional dendritic cell inoculation, splenic MDSCs abundance decreased after two treatments with 5-FU (50 mg/kg) [75].

The role of DOX and 5-FU has since been explored in TNBC models. The effect of DOX (2.5 mg/kg and 5 mg/kg) and 5-FU (50 mg/kg) on MDSCs was studied in a 4T1 TNBC orthotopic mouse model [76]. There was a dose-dependent reduction in intertumoral MDSCs in DOX-treated mice, however neither reduction was statistically significant. Furthermore, only 5 mg/kg DOX was able to statistically reduce splenic MDSCs. Meanwhile, 5-FU decreased both intratumoral and splenic MDSCs in a statistically significantly manner [76].

Meanwhile another orthotopic 4T1 study showed slightly different results [28]. Mice were treated with either 2.5 mg/kg or 5 mg/kg I.P. of DOX on day 7 and 12 and tissues were collected on day 14, 17 and 23 [28]. Both 2.5- and 5 mg/kg DOX decreased MDSCs in spleen and blood 48 h after the second treatment. However, the levels of MDSCs went back up in the blood for 2.5 mg/kg after 2 days and after 5 days for 5 mg/kg. This study confirmed that the immune response of DOX is dose-dependent as well as highlighting its temporal nature.

Lastly, 5-FU was given I.P. at 5, 10, 25 or 50 mg/kg three times a week for 3 weeks to orthotopic 4T1 tumour-bearing mice [31]. Similarly, there was a decrease in circulating MDSCs, observed all 3 weeks, when treated with 5-FU 50 mg/kg. Interestingly, all other doses showed no change except 10 mg/kg which showed an increase in circulating MDSCs at week 3. This seemingly random increase could be due to biological variability; however, the authors d0 not address these changes [31].

### 4.3. Cyclophosphamide (CTX)

On the other hand, CTX has been shown to increase MDSC abundance. In a ret transgenic skin tumour mouse model, 1 mg/mouse I.P. of CTX decreased T-regs in the spleen, blood and tumour while increasing MDSCs systemically but not in the tumour. Meanwhile, 2.5 mg/mouse I.P. of CTX also decreased T-regs but increased the abundance of MDSCs both systemically and in the tumour [77]. Similarly, in a SW1C K1735 subcutaneous melanoma mouse model, one dose of 2 mg/kg I.P. CTX increased MDSCs [78]. However, in a TC-1 subcutaneous lung epithelial mouse model, while CTX showed an increase in splenic MDSCs after a single 50 mg/kg dose, daily doses of 10 mg/kg did not affect splenic MDSCs levels [79]. Interestingly while CTX, dissolved in drinking water, increased circulating MDSCs in non-tumour-bearing Balb/C mice, there was no increase seen in 4T1 TNBC tumour-bearing mice at varying concentrations (5–40 mg/kg) [31].

### 4.4. Taxanes

Mice bearing a 4T1-Neu subcutaneous tumour were treated with 33 mg/kg I.P. docetaxel twice, 7 days apart [80]. It was seen that docetaxel reduced MDSCs, which will be explored further in the next section. Chemotherapies can kill MDSCs or can differentiate them into macrophages. Unlike the other studies discussed, it was shown that docetaxel increased M1-like and decreased M2-like markers in vivo. Furthermore, when MDSCs were purified from tumour-bearing mice and cultured with docetaxel, M1-like macrophage markers levels were higher than untreated MDSCs.

### 4.5. Further Classification of MDSCs

While CD11b+ GR1+ has been used to quantify MDSCs in the aforementioned studies, more recent research has shown that this is too general of a marker as subpopulations have been discovered [81]. There are two major classifications of MDSCs, granulocytic or polymorphonuclear (G, PMN) and monocytic (M) MDSCs. Expanding our knowledge on MDSC subpopulations allows us to better understand chemotherapies’ role on MDSCs.

A 2016 study evaluated GEM’s effect on circulating immune cells during treatment of pancreatic cancer patients [39]. GEM was given at day 1, 8 and 15 in a 28-day treatment cycle. In this study patients’ blood was drawn before each treatment and on day 29 before the next treatment cycle. GEM was shown to reduce granulocytic MDSCs (CD11b+ CD14-CD33+ HLA-DR-) while there was no change in monocytic MDSCs (CD11b+ CD14+ CD33+ HLA-DR-) [39]. More specifically to TNBC, a 2020 study treated E0771 subcutaneous (flank) tumour-bearing mice with either 60 mg/kg GEM I.P. on days 10, 14, 18 and 22 or a single treatment on day 22 [82]. Tissues were collected 48 h after the last treatment (day 24). After a single treatment of GEM, splenic and tumour M-MDSCs (Ly6C^high^) abundance was decreased. However, repeated treatment increased splenic M-MDSCs and had no impact on tumour M-MDSCs [82]. A 2024 study treated 4T1 subcutaneous (flank) tumour-bearing mice with 60 mg/kg I.P. GEM three times over 3 weeks and collected tissues 24, 48 or 96 h after the last treatment [44]. GEM reduced splenic granulocytes (CD11b+ Ly6G+), monocytes (CD11b+ Ly6G-F4/80-Ly6C+), macrophages (CD11b+ Ly6G-Ly6C-F4/80+), and inflammatory monocytes (CD11b+ Ly6G-F4/80+ Ly6C^high^). In the tumour, GEM was only effective in reducing the inflammatory monocyte population. It was demonstrated that the reduction in these populations was acute, seeing less reduction over time [44]. In our studies, using a 4T1 orthoptic (mammary-fat pad) mouse model, we saw that GEM (60 mg/kg) significantly reduced both splenic and tumour M-MDSC abundance (Cd45+ CD11b+ Ly6G-Ly6Chigh) [45]. Interestingly, we saw a small decrease in splenic pan-MDSCs (CD45+ CD11b+ GR1+) and a significant decrease in splenic granulocytic MDSCS (CD45+ CD11b+ Ly6G+ Ly6C-) with a statistically significant increase in tumour pan-MDSCs and granulocytic MDSCs. While these changes were statistically significant, they may not be biologically relent as the % changes were quite small. Again, we saw that the reduction in M-MDSCs was dose-dependent with 60 mg/kg having a greater effect than both 30 and 15 mg/kg. Furthermore, we showed that 20 mg/kg I.V. GEM changes both T-cells, macrophages and MDSCs in the same fashion as 60 mg/kg I.P GEM [45].

The effect of Paclitaxel on both M-MDSCs (CD11b^+^HLA-DR^low/−^CD14^+^CD15^−^) and PMN-MDSCs (CD11b^+^HLA-DR^low/−^CD14^−^CD15^+^) was measured in both the blood and tumour and correlated to response of nine metastatic melanoma patients (NCT02332642) [83]. Patients who responded to treatment had higher M-MDSC levels before treatment but a decrease in blood and tumour M-MDSCs levels was seen post-treatment. However, in patients who did not respond, MDSC levels tended to increase [83]. In a metallothionein-I/ret transgenic melanoma mouse model, 1 mg/kg paclitaxel I.P. was given once every 3 weeks. While paclitaxel induced no change in MDSCs (CD11b+ Gr1+) in the metastatic lymph nodes, spleen or blood, there was a significant reduction in tumour MDSCs [84]. This is similar to what was seen in a 4T1 TNBC model [29]. Paclitaxel (30 mg/kg or 75 mg/kg) was loaded into micelles (POx-PTX) and given I.V. every 4 days for 2 weeks. POx-PTX reduced G-MDSCs (CD45+ CD11b+ Ly6G+) while not changing M-MDSC (CD45+ CD11b+ Ly6C+) abundance [29]. Again, chemotherapies behave differently between cancer types and the evaluation of each chemotherapy in TNBC is needed.

## 5. Impact of Chemotherapy on Other Immune Cells

The bulk of research, and our review, have focused on the impact of chemotherapy on T-cells, macrophages and MDSCs; however, it is also important to highlight investigations that also focus on less abundant immune populations while being cognizant of the limited number of studies. In an in vitro experiment, three different breast cancer cells (MCF-7 (ER+), SKBR-3 (HER2+) and MDA-MB-231 (TNBC)) were treated with either NK cells, EPI or a pretreatment of EPI and then NK cells [85]. It was seen that pretreatment with EPI enhanced the cytotoxicity of NK cells on breast cancer cells. In an orthotopic 4T1 study mice were treated with either 2.5 mg/kg or 5 mg/kg I.P. of DOX on day 7 and 12 and tissues were collected on day 14, 17 and 23 [28]. NK abundance increased in the blood in a dose-dependent manner; however, there was no change in abundance in the spleen. Like the T-cell results previously discussed in Section 2.1, this increase in NK cells only lasted 5 days after treatment, where levels became same as untreated mice 10 days after treatment. While there seems to be a benefit of chemotherapy in increasing NK cytotoxicity, breast cancer patients’ levels of NK cells are severely affected when treated with NAC. Breast cancer patients saw a significant drop in NK and B-cells 2 weeks after NAC, and even after 9 months levels were still below pretreatment baseline [86]. More specifically, peripheral blood was taken from 56 TNBC patients receiving NAC (albumin-bound paclitaxel Nab-Pac, EPI and CTX), and the immune cell abundance was quantified [87]. After the first phase of treatment, there were no significant changes in immune cell abundance, however after the second phase, B-cell and NK abundance dropped to 10% and 50% respectively. CD4+ T-cells also dropped to 50%, while CD8+ abundance was not affected. After the first phase of treatment, there were no significant changes in immune cell abundance; however, after the second phase, B-cell and NK abundance dropped to 10% and 50% respectfully. CD4+ T-cells also dropped to 50%, while CD8+ abundance was not affected. In another breast cancer study no change in circulatory NK abundance was seen after eight cycles of NAC for partial or poor responders [88]. However, patients who elicited a very good response of pCR had a significant increase in circulating NK cells compared to pretreatment levels. Interestingly, the % of NK cells returned to pretreatment levels after surgery for poor and good responders. Whether before or after surgery, those who achieved pCR had higher NK levels than healthy donors [88]. While there is less literature exploring these immune cell types in TNBC, much work has been done in other cancers. The role of chemotherapy on NK [89] and B-cells [90] is very complicated and varies between drugs and cancer types, similarly to what we have explored with T-cells, macrophages and MDSCs.

## 6. Conclusions

While chemotherapy has become the backbone of TNBC treatment, there is still a large population that does not achieve clinical benefit. With the growing interest in immunotherapies for the treatment of TNBC, it is essential to understand the impact of chemotherapies on the immune system to deliver the best possible combination therapies. This review has highlighted the complexity of chemotherapies’ abilities to modulate the immune system. It is first seen that the effectiveness of chemotherapy in increasing pro-inflammatory cells or decreasing anti-inflammatory cells changes between cancer types. Next, it was seen that the efficacy changed based on concentration and dosing frequency. Lastly, and maybe most importantly for combination therapies, is that the response was temporal in nature. While data was available for many of the chemotherapies, there is still a large gap in their function in TNBC specifically. Furthermore, the TNBC studies mostly used 4T1 models which does not fully represent the high heterogeneity of TNBC. We recommend using alternative syngeneic models such as AT3 and Py8119 which would diversify the immune profile being explored, especially since these tumours are from C57BL/6 mice. We also recommend using humanised TNBC PDX models or GEM models such as C3(1)-Tag that allow a more “natural” immune evolution. By diversifying the TNBC models used, the translatability of these therapies will greatly improve. However, we do believe that this review has highlighted that chemotherapy can be used to modulate the immune system and prime the TNBC TME for further treatment with immunotherapies. Lastly, we suggest that it may be possible to further improve response rates by selecting which approved chemotherapy TNBC patients receive by based on their immune abilities that match patient’s immune phenotypes (i.e., high MDSC, low T-cell). In saying this, we are aware that routine immune profiling is not performed except possibly TILs [91]. We urge the development and use of standardised protocols in the clinic for the quantification of immune populations, similar to the ones being used in research settings such as OMIP-102 [92]. While there is much work to be done in fully understanding chemotherapies’ immune roles, their use as immune modulators will be a key in the future of TNBC treatment.

## Figures and Tables

**Table 1:** List of chemotherapies used either first-line or in metastatic TNBC. Table includes chemotherapies mechanism of action and in which combination or patients it is used in.

Chemotherapy	Mechanism	First-Line	Metastatic	Patient
5-fluorouracil (5-FU)	Antimetabolite (pyrimidine analogue)	+	Rare	FEC regime
Epirubicin	Anthracycline (topoisomerase II inhibitor)	+	Rare	FEC regime
Doxorubicin (DOX)	Anthracycline (topoisomerase II inhibitor)	+	Select	AC-T regime
Cyclophosphamide (CTX)	Alkylating agent	+	Rare	AC-T and FEC regime
Taxanes: paclitaxel (PTX)	Microtubule stabiliser	+	+	
Platins: carboplatin	DNA cross-linking platinum agent	+	+	Used in BRCA+ patients (20% of TNBC)
Gemcitabine (GEM)	Antimetabolite (pyrimidine analogue)	-	+	Used in combination (PTX and carboplatin)

**Table 2:** List of immune cells and the markers used for detection in flow cytometry and IHC. Table includes the immune cells most common tumorigenicity status in the context of TNBC.

Lineage	Immune Cell	Flow Cytometry/IHC Detection Marker	TNBC Tumorigenicity Status
Lymphocyte	General T-cell	CD3+	Context-dependent
	Cytotoxic T-cell	CD8+	Anti-Tumourigenic
	T-helper (Th)	CD4+	Context-dependent
	T-regulatory (Treg)	CD4+ FOXP3+ CD4+ CD25+	Pro-Tumourigenic
	T-regulatory & Cytotoxic	Tumour Necrosis Factor Receptor 2 (TNFR2+)	Context-dependent
	gd T-cells	gd TCR	Anti-Tumourigenic
	Exhausted T-cell	Relevant T-cells marker + Programmed Death 1 (PD-1)	Pro-Tumourigenic (immune evasion)
Monocyte	Monocytes	CD11b+	Pro-Tumourigenic (Typically)
	Unpolarised Macrophages (M_o_)	F4/80+	Variable
	M2-like Tumour-Associated Macrophages (TAMs)	CD163+, CD206+, Arg1+	Pro-Tumourigenic
	M1-like Tumour-Associated Macrophages (TAMs)	CD80+, MHCII+	Anti-Tumourigenic
	Myeloid-Derived Suppressor Cells (MDSCs)	GR1+	Pro-Tumourigenic
	Monocytic MDSCs	GR1+, Ly6C+	Pro-Tumourigenic
	Granulocytic MDSCs	Ly6G+	Pro-Tumourigenic

## Data Availability

No new data were created or analysed in this study. Data sharing is not applicable to this article.

## Abbreviations

The following abbreviations are used in this manuscript:

5-FU	5-fluorouracil
ARG1	arginase 1
BC	breast cancer
CCR7	C-C Chemokine Receptor type 7
CTLA-4	cytotoxic T lymphocyte-associated protein-4
CTX	cyclophosphamide
DFS	disease-free survival
DOX	doxorubicin
EPI	epirubicin
ER	oestrogen receptor
G-MDSCs	granulocytic myeloid-derived suppressor cells
GEM	gemcitabine
HER2	human epidermal growth factor receptor 2
ICI	immune checkpoint inhibitor
I.P.	intraperitoneal
I.V.	intravenous
IFN-γ	Interferon Gamma
IL,4,6,8,10,17	Interleukin 4,6,8,10,17
iNOS	inducible Nitric Oxide Synthase
M-MDSCs	monocytic myeloid-derived suppressor cells
MDSC	myeloid-derived suppressor cells
Nab-PTX	albumin-bound paclitaxel
NAC	neoadjuvant chemotherapy
NO	nitric oxide
OS	overall survival
pCR	pathological complete response
PD-L1	programmed death ligand 1
PD1	programmed cell death protein 1
PMs	peritoneal macrophages
POx-PTX	paclitaxel micelles
PR	progesterone receptor
PTX	paclitaxel
TAMs	tumour-associated macrophages
TILs	tumour-infiltrating lymphocytes
TIM-3	T-cell immunoglobulin and mucin domain-3
TME	tumour microenvironment
TNBC	triple-negative breast cancer
TNFα	Tumour Necrosis Factor Alpha
